# The Impact of Iodine Concentration Disorders on Health and Cancer

**DOI:** 10.3390/nu14112209

**Published:** 2022-05-26

**Authors:** Mateusz Winder, Zofia Kosztyła, Aleksandra Boral, Piotr Kocełak, Jerzy Chudek

**Affiliations:** 1 Department of Radiology and Nuclear Medicine, Medical University of Silesia, 40-752 Katowice, Poland; 2 Students’ Scientific Society, Department of Radiology and Nuclear Medicine, Medical University of Silesia, 40-752 Katowice, Poland; zkosztyla@gmail.com (Z.K.); boral-a@wp.pl (A.B.); 3 Department of Pathophysiology, Medical University of Silesia, 40-752 Katowice, Poland; pkocelak@sum.edu.pl; 4 Department of Internal Medicine and Oncological Chemotherapy, Medical University of Silesia, 40-027 Katowice, Poland; chj@poczta.fm

**Keywords:** iodine, iodine deficiency, prevalence, iodine supplementation, thyroid dysfunction, cancer

## Abstract

Iodine deficiency is an ongoing problem. The implementation of salt iodization has significantly reduced the effects of iodine deficiency worldwide in recent years, and the remaining iodine deficiency is mild to moderate. Iodine is an essential substrate for the synthesis of thyroid hormones in the thyroid gland. It can also act as an antioxidant, as well as an anti-proliferative and pro-apoptotic factor. Pregnant women, breastfeeding women, and children are particularly affected by iodine deficiency. It leads to thyroid diseases and metabolic and developmental disorders, as well as cancer. However, an excessive iodine intake may, similarly to iodine deficiency, lead to the development of goiter, and toxic amounts of iodine can lead to thyroiditis, hyperthyroidism, and hypothyroidism, and even to the development of papillary thyroid cancer. Correcting iodine deficiency potentially reduces the chance of developing malignancies. Additional research is needed to better understand both the effect of iodine on carcinogenesis and the clinical outcome of iodine deficiency compensation on cancer patients’ prognosis. The upcoming public health challenge appears to be reducing salt consumption, which could result in a lower iodine intake. Thus, an iodine enrichment vehicle other than salt could be considered if salt iodine levels are not increased to compensate, and urine iodine levels should be monitored more frequently.

## 1. The Source of Iodine

Iodine (I) is a chemical element from the group of halogens with an atomic mass of 53 that occurs in nature as iodide. A significant amount of this element is found in oceans, where its concentration is approximately 50 μg/L. From seawater, iodide is oxidized to elemental iodine by a photochemical process with the participation of atmospheric ozone and via phytoplankton and seaweed. The iodine then evaporates into the atmosphere and returns with the rainfall as aerosols or gaseous forms, reaching the surface of the soil and plants [1]. This circulation in many regions of the world may be disturbed; therefore, drinking water and soil contain traces of this element. This results in lower iodine levels in crops and, therefore, the same food products from different regions of the world may contain different amounts of iodine. The areas with iodine deficiency are mainly mountain areas, areas with frequent flooding, and many inland regions of Central Asia, Africa, Central, and Eastern Europe [2]. In humans, iodine is both a micronutrient and an essential nutrient, where its deficiency causes many abnormalities [3]. The amount of iodine ingested with food is proportional to its presence in the environment and diet [4]. The natural sources from which iodine is obtained are seawater, seaweed, and saltpeter deposits. It is estimated that over one third of the world’s population is exposed to iodine deficiency, especially in mountainous areas [5]. The most important sources of iodine in the diet are seafood, eggs, and dairy products (partly due to the use of iodine and iodophors disinfectants in the dairy industry) [6]. Iodine-rich foods also include cod, pollock, salmon, wheat bran, broccoli, dry pea seeds, and hazelnuts [7]. The most abundant natural source of iodine is seaweed [6]. However, nowadays, the main source of iodine in areas of deficiency is iodized salt. The fortification of food products with iodine, including salt iodization, has contributed to the reduction in the incidence of the once common goiter and hypothyroidism. Despite the common indications for salt iodization, in some countries, the salt contained in processed products is non-iodized [8].

## 2. Application of Iodine in Medicine

The iodine element is widely used in medicine, as well as in the food industry. Large amounts of iodine are used to prevent its deficiency. The main method of iodine fortification is salt iodization, which is a common practice around the world. In addition, iodine is an active ingredient of drugs and a component of substances used in imaging examinations, and its isotopes are a carrier of radioactive energy.

Iodine 131 and 123 isotopes are used in thyroid scintigraphy to assess thyroid function and detect metastases [9]. Scintigraphy is also important in assessing the prognosis in patients with heart failure, where the catecholamine analog, 123I-meta-iodobenzylguanidine (MIBG), is employed [10]. Radioiodine therapy is an effective treatment in the case of hyperthyroidism in patients with Graves’ disease, toxic adenoma, and toxic multinodular goiter, and in patients with well-differentiated thyroid cancer [11]. The radioactive iodine is taken up and accumulated by the hyperactive thyroid gland or by differentiated thyroid tumors. A high concentration of radioactive iodine destroys the tissues of the thyroid gland and reduces the production of hormones.

Iodine is also found in drugs, including class III anti-arrhythmic Amiodarone (75 mg of iodine in one 200 mg tablet), the use of which may cause both hypo and hyperthyroidism [12]. Oral and intravenous (i.v.) iodine contrast agents containing 140–400 mg of iodine in 1 mL are commonly used in imaging diagnostics, especially in computed tomography (CT) and angiography. The average amount of iodine-contrast administered i.v. is approximately 1 mL per 1 kg of body weight. This means that a person weighing 75 kg can receive approximately 30 g of iodine during one examination. Their administration is contraindicated in overt hyperthyroidism and severe chronic kidney disease (CKD) [13]. 

Other drugs containing iodine are Lugol’s solution (127 mg of iodine in 1 mL) and povidone-iodine, which is used as a disinfectant (10 mg of iodine in 1 mL) [12,14]. Potassium iodide (KI) is also an ingredient in topical eye drops to treat opacities in the vitreous body and lens or as an antiseptic [15]. Potassium iodide is also used in dermatology in the treatment of dermatoses as a first or second choice drug [16]. Moreover, iodine is used as a food preservative [17].

## 3. The Biological Role of Iodine

Iodine is a trace element that is absorbed from food in the stomach and duodenum. In healthy adults, over 90% of ingested iodine is absorbed as potassium iodide [18]. Subsequently, the iodine in the plasma is either taken up, mainly by the thyroid gland, or excreted by the kidneys.

The renal iodine clearance is fairly constant, whereas the thyroid clearance varies adaptively with iodine intake and plasma concentration [19]. In areas with a sufficient amount of iodine in the environment, the average daily turnover of iodine through the thyroid gland is approximately 60–95 µg in adults. When the iodine supply is adequate, no more than 10% of any absorbed iodine is taken up by the thyroid gland [4]. However, when its dietary intake is low, the thyroid gland uptake of iodine increases. In chronic iodine deficiency, this fraction may exceed 80% [20].

The body of a healthy adult human contains 15–20 mg of iodine, 70–80% of which is found in the thyroid gland. In chronic iodine deficiency, its content in the thyroid gland may drop below 20 μg. The thyroid gland of the majority of animal species contains an amount of thyroid hormones large enough to cover the body’s needs for 2 to 10 weeks, provided that the iodine intake was sufficient beforehand [20]. More than 90% of the ingested iodine is excreted in the urine, whereas only a small amount appears in the stool [4].

Iodine is an essential substrate for the synthesis of thyroid hormones in the thyroid gland. The sodium–iodine symporter (NIS), found in the basal membrane of thyroid cells, actively transports iodides to the thyroid cells over a concentration gradient 20–50 times higher than in plasma. On the thyroid surface, the enzymes thyroperoxidase (TPO) and hydrogen peroxide oxidize iodide and then attach it to the tyrosyl residues on thyroglobulin, forming monoiodothyrosine (MIT) and diiodothyrosine (DIT)—precursors to thyroid hormones. TPO catalyzes the coupling of the phenyl groups of iodothyrosines through the diether bridge, creating the thyroid hormones thyroxine (T4) and triiodothyronine (T3) [21]. Iodine is released back into the plasma by the tissue degradation of free forms of thyroid hormones (fT4 and fT3). The half-lives of fT4 and fT3 are 5–8 and 1.5–3 days, respectively. The iodine can then be either taken up by the thyroid gland or excreted via the kidneys [22].

Thyroid hormones are important for the proper development of tissues and organs and the control of the metabolic rate. T4 is the main secretory product of the thyroid gland: its daily secretion is approximately 80 μg, whereas T3 is secreted in the amount of approximately 4–6 μg/day and is mainly produced as a result of conversion from T4, which is a prohormone. In tissues, T4 is deiodinated in the 5 ′position to T3 with the participation of type 1 (DIO1) or type 2 (DIO2) deiodinase enzymes or to the metabolically inactive rT3 (reverse T3) under the influence of DIO1 or type 3 deiodinase (DIO3) [21]. Thyroid hormones play a major role in the development of the central nervous system (CNS) of humans from 15 weeks of gestation to 3 years of age. Their deficiency during this period leads to irreversible malformations. However, fetal brain development is vulnerable to iodine deficiency even earlier, prior to 14 weeks of gestation [23]. Thyroid hormones also control the rate of metabolism of carbohydrates, proteins, vitamins, fats, and minerals [24].

Iodine, in addition to being a component of thyroid hormones, can also act as an antioxidant, as well as an anti-proliferative and pro-apoptotic factor. It also has an antibacterial and anti-inflammatory effect by neutralizing radical oxygen species [25,26].

Iodine is also accumulated by the mammary gland and excreted into breast milk so that this element is available to the baby [24]. Small amounts of iodine are also taken up by the salivary glands, gastric mucosa, and choroidal plexuses.

## 4. Measurement of Iodine Body Resources

The best method of determining the iodine body resources is the measurement of iodine in the urine using the Sandell–Kolthoff reaction catalyzed by iodine or the inductively coupled plasma mass spectroscopy (ICP-MS) [27]. On this basis, the daily demand for this element is determined. According to the findings of the International Commission for Iodine Deficiency Disorders, the minimum normal urinary iodine concentration should be 0.79 µmol/L, which corresponds to the daily requirement of approximately 150 µg [28]. In children and non-pregnant women, median urinary iodine concentrations of between 100 μg/L and 299 μg/L define a population that has no iodine deficiency. In addition, not more than 20% of samples should be below 50 μg/L. Nevertheless, the study by Xiu et al. [29] proposed an upper limit for the urinary iodine concentration of 200 µg/L. This was due to the increased incidence of thyroid goiter at urine iodine concentrations both below 20 µg/L and above 200 µg/L in school-aged children. In non-pregnant and non-lactating women, a urinary iodine concentration of 100 μg/L corresponds to a daily iodine intake of approximately 150 μg. During pregnancy, appropriate median urinary iodine concentrations should be between 150 μg/L and 249 μg/L [28]. The population norms proposed by the World Health Organization (WHO) for urinary iodine concentrations and the grades of iodine deficiency are presented in (Table 1) [28,30]. 

The severity of iodine deficiency corresponds to the prevalence of goiter in children, which is 0–4.9% in the absence of iodine deficiency, 5–19.9% in mild deficiency, 20–29.9% in moderate deficiency, and >30% in severe deficiency [28].

The urinary excretion of iodine is believed to be the most sensitive and reliable method for assessing iodine intake in a given population. This is due to the fact that approximately 90% of the ingested iodine is excreted by the kidneys. The test is performed with a morning or random urine sample. The reliability of the results is similar to the results obtained in the case of the daily urine collection or the iodine excretion to creatinine index. The iodine content in urine reflects its intake over the previous hours to days [31]. For population iodine measurements, it is impractical to collect 24 h urine samples; therefore, single urine samples are collected so that, when expressed as a median, they correlate with the 24 h samples, despite variations in hydration status and changes overnight [32]. However, individual iodine intake should be estimated based on at least 10 repeated spot or 24 h urine samples (excretion of iodine in the urine), or by the iodine-to-creatinine ratio, taking into account the fact that low creatinine levels may be due to malnutrition [33,34]. In a population study, the daily iodine intake can be estimated from urinary iodine measurements in the mean daily urine volume, assuming that 92% of iodine is excreted in the urine [urinary iodine (ug/L) × 0.0235 × body weight (kg) = daily iodine intake [35]. 

Other methods recommended for the population iodine assessment are the goiter rate, serum thyroid-stimulating hormone (TSH), and serum thyroglobulin (Tg). 

The measurement of goiter is carried out by the methods of palpation and ultrasound; however, due to the unsatisfactory sensitivity and specificity of palpation in iodine-deficiency areas, the ultrasound method for the measurement of the thyroid volume is preferable [36]. In populations with an increasing iodine supplementation, the thyroid volume reflects the previous iodine deficiency and current status, since the regression of thyroid volume may not occur completely even after the initiation of salt iodination [37]. Thus, for the actual assessment of the thyroid gland volume, standardized and up-to-date recommendations are necessary, based on measurements made in school-age children in iodine-sufficient areas [38]. The goiter incidence rate is used to reflect the long-term supply of iodine (from months to years). Some parameters of the ultrasound are better at assessing the change in goiter (body volume/surface area) [39].

Circulating TSH reflects the iodine intake: elevated TSH may be due to iodine deficiency in older children and adults; however, TSH levels are relatively insensitive to iodine nutritional status in adults [32]. Nevertheless, TSH reflects the iodine status of newborns [40,41]. TSH levels >5 mIU/L in more than 3% of neonatal blood samples collected 3–4 days after birth indicate iodine deficiency in the population.

The Tg concentration is higher in endemic goiter regions due to TSH stimulation and a higher cell mass, and both iodine deficiency and excess. The preferred method of assessing the concentration of Tg is its measurement in dry blood spot (BDS), as this method is easier to perform and requires minimal amounts of blood compared to standard serum assays [42].

## 5. The Effects of Iodine Deficiency and Excess

The recommended daily allowance (RDA) of iodine is 150 µg for adults, 220 to 250 µg for pregnant women, and 250 to 290 µg for breastfeeding women [28,43].

### 5.1. Iodine Deficiency

Iodine deficiency has many adverse effects on tissue development due to its key function in the synthesis of thyroid hormones [2]. Nonetheless, even when the iodine intake is quite low, the thyroid hormone production is normal. Hypothyroidism in adults does not occur until the daily iodine intake is below approximately 10 to 20 μg, which is approximately 10–20% of the average iodine intake in the US population [44]. 

Supplying too little iodine in the diet leads to the insufficient synthesis of thyroid hormones. It affects the functioning of many organs, especially the muscles, heart, liver, and kidneys. This leads to medical conditions called iodine deficiency disorders (IDDs). Iodine deficiency in the diet results in an increased secretion of TSH, which stimulates the thyroid tissue, causing its hypertrophy. A long-term iodine intake of less than 50 µg/day usually leads to thyroid goiter. Severe and long-term iodine deficiency may lead to hypothyroidism [45].

In pregnant women, iodine deficiency may result in severe neurological deficits and the formation of goiter in their offspring. Lower degrees of iodine deficiency may also cause significant neurodevelopmental deficits in infants and children [44,46]. The most serious consequence of iodine deficiency is damage to the fetus. Maternal thyroxin crosses the placenta before the onset of thyroid function in the fetus at week 10–12 and accounts for up to 20–40% of T4 measured in umbilical cord blood at birth [47]. Optimal amounts of thyroid hormones are needed for the migration of neurons and the myelination of the fetal brain; therefore, insufficient iodine irreversibly disrupts brain development [48]. Severe iodine deficiency during pregnancy increases the risk of stillbirths, miscarriages, birth defects, perinatal morbidity, and mortality, and can cause significant mental retardation, along with a short stature, mutism, and spasticity [49,50,51,52,53]. In areas with a very low iodine content, mental retardation may affect up to 5–15% of newborns. The most severe mental form of iodine deficiency is overt cretinism, which, together with a milder degree of intellectual deterioration, was the leading cause of preventable mental impairment in 1990 [54]. 

In children and adolescents with iodine deficiency, a delay in physical development and mental impairment are observed, whereas, in adults, iodine deficiency may result in apathy secondary to hypothyroidism and a reduced work efficiency [4]. Iodine deficiency causes goiter development. If it occurs in >5% of children 6–12 years of age, it is called endemic goiter. The microscopic image of the goiter is an overgrowth that does not differ from the changes occurring in the sporadic or simple goiter. However, recurrent episodes of hyperplasia are followed by the processes of involution and atrophy, known as the Marine cycle [55]. The thyroid gland is usually diffuse in children and often nodular in adults. A typical biochemical presentation is normal or elevated TSH levels, normal or low fT4 levels, normal or elevated T3 levels, and the absence of anti-thyreoperoxidase or anti-thyroglobulin antibodies.

To maintain the normal secretory function of the thyroid gland, adaptive mechanisms are activated, such as an increased TSH secretion, increased sensitivity of follicular cells to TSH, increased thyroid iodine concentration, preferential T3 secretion, and thyroid hyperplasia [56,57]. The critical iodine threshold of 50 µg per day is sufficient for the organic iodine content in the thyroid gland and the production of thyroid hormones, but, below this value, the mechanisms become insufficient and the iodine content in the thyroid decreases, causing the development of goiter [58].

Iodine insufficiency seems to be a risk factor for thyroid cancer development, particularly follicular carcinoma [59]. The main plausible mechanism is an increased TSH concentration and the enhancement of cellular proliferation [59]. Moreover, it was reported that, in countries with a high iodine intake, the ratio of PTC: FTC (papillary to follicular thyroid cancer) was higher than in countries with a lower iodine intake. It seems that iodine deficiency may be a weak initiator of thyroid cancer and a strong promoter of carcinogenesis in animal studies. In areas of a high iodine supply, there are fewer aggressive follicular and anaplastic thyroid cancers and more papillary cancers. After the introduction of iodine prophylaxis, a change in the prevalence of thyroid cancers appears towards less malignant thyroid cancers [60].

In all age categories, iodine deficiency may result in an increased susceptibility of the thyroid to nuclear radiation, especially in children [32].

### 5.2. Excess Iodine

The excessive consumption of iodine can lead to its accumulation in the body and the resulting side effects, including poisoning. The effects of excess iodine are caused by a disturbance in the function of the thyroid gland, and may vary. Excess iodine may result in subclinical or overt thyroid dysfunction, especially in patients with risk factors such as pre-existing thyroid disease and in the elderly [17,61]. An excessive iodine intake is one of the main risk factors for Hashimoto thyroiditis [62]. 

The effects of iodine excess may also result from corrections of iodine deficiency. Both low and high iodine supplies can impair thyroid function. The range of daily iodine intake between 600 and 1100 µg/day is well tolerated in subjects with normal thyroid function [35]. The prolonged duration of iodine deficiency leads to the lowering of the upper limit of iodine tolerance [63].

There has been a significant increase in the number of imaging diagnostic examinations utilizing i.v. contrast enhancement in recent years [64,65]. High levels of iodine contained in iodine-based contrast media may lead to thyroid dysfunction according to the Jod–Basedow effect [65,66,67]. The prevalence of both contrast-induced hyper- and hypothyroidism is estimated at 1–15% [66]. Exposure to iodinated contrast media before or during pregnancy may also increase the risk of thyroid dysfunction in newborns [68]. Although most cases of iodine contrast-induced hyperthyroidism are mild and transient, there is a small risk of severe thyrotoxicosis with serious cardiovascular complications, especially in elderly patients [66]. Also worth mentioning is acute kidney injury (AKI), which is a clinically significant complication after the i.v. use of iodinated contrast agents, the so-called contrast-induced nephropathy (CIN). It is a common cause of hospital-acquired AKI and is associated with an increased mortality [69]. However, it is not a result of an overdose of iodine, but a reduction in blood flow in the renal medulla.

Excess iodine may give rise to thyroiditis, hyperthyroidism, and hypothyroidism, and even to the development of papillary thyroid cancer [70]. It has been shown that iodine overdose may lead to irreversible damage to the retinal pigment epithelium and photoreceptors [71,72]. Acute iodine toxicity is rare and often difficult to diagnose. Non-specific clinical symptoms may include nausea, vomiting, and diarrhea, as well as delirium, dementia, and shock [72]. It is estimated that the consumption of 2 ÷ 4 g of iodine may cause death, and the few reported fatal cases of iodine poisoning after the ingestion of iodine-containing compounds concerned doses of 17–120 mg/kg [73,74]. 

An excessive iodine intake may, similarly to iodine deficiency, lead to the development of goiter. A diet rich in seaweed resulted in endemic goiter in Northern Japan with the excretion of iodine exceeding 20 mg/day [75]. Similar reports also concerned mainland and coastal China [76,77]. Moreover, the rate of subclinical hypothyroidism increases due to thyroid autoimmunity and the inhibition of hormone synthesis (the Wolff–Chaikoff phenomenon) [78]. 

The main complication of iodine prophylaxis is iodine-induced hyperthyroidism (IHH). It occurs in many supplementation programs but can hardly be avoided even by the strict monitoring of the iodine supply [79,80]. The risk of the development of hyperthyroidism was associated with a rapid increase in iodine intake and iodine overload in long-lasting iodine-deficient African countries [81]. However, an increase in the hyperthyroidism incidence was also observed in Switzerland after only a slight increase in the iodine supply after iodine salt fortification (a 27% increase in incidence within one year after an increase in the iodine supply from 90 to 150 µg/day) [82]. The main reason for the development of hyperthyroidism after iodine fortification is the development of multifocal autonomous growth with cells that acquired the activation mutations of the TSH receptor as a result of iodine deficiency [83]. This may be considered a complication of iodine deficiency, especially in populations with moderate to severe iodine deficiency. Most often, symptomatic patients were over 40 years of age, with multinodular goiter and cardiovascular disease [84]. One to ten years after starting iodine supplementation, the incidence of hyperthyroidism drops to a level even lower than the initial level [85].

Population studies have shown an increased incidence of thyroiditis in the US population after the initiation of iodine salt enrichment and the development of anti-thyroid antibodies in the Greek population after the introduction of iodized oil [86,87]. The population with a higher iodine intake has a higher incidence of anti-thyroid antibodies and hypothyroidism [88,89,90,91,92]. An increased iodine intake causes the development of autoimmune thyroiditis, potentially by increasing the immunogenicity of thyroglobulin and damaging thyroid cells with free radicals.

The introduction of iodine fortifications programs may also result in the development of hypothyroidism. The data from the Danish population revealed an increase in overt hypothyroidism in a 7-year follow-up after the initiation of salt iodization [93,94]. New cases of hypothyroidism occurred mainly in young subjects with a previous iodine deficiency. Hypothyroidism is the consequence of a chronic high iodine intake since iodine has an inhibitory effect on thyroid hormone synthesis and secretion [95,96]. The second mechanism responsible for hypothyroidism is related to the development of autoimmune thyroiditis [97]. Histological studies have shown an increased incidence of thyroiditis in goiter after the initiation of iodine prophylaxis [98].

Three days after the Chernobyl disaster in 1986, potassium iodide prophylaxis was implemented in Poland to minimize the negative effects of radioactive iodine released into the atmosphere. This action was based on the principle of the Wolff–Chaikoff phenomenon. A large dose of iodine contained in Lugol’s solution blocked the uptake of the radioactive element by the thyroid gland. This allowed for the minimization of the risk of developing thyroid cancer. However, from 1987 to 1997, the increase in the prevalence of differentiated thyroid cancer in the Polish adult population, especially in women over 40 years old, was observed [99]. The recommended dosing regimens for KI Lugol’s solution were 15 mg for neonates, 50 mg for children up to 5 years of age, and 70 mg for all minors. KI prophylaxis was not recommended for adults, except for pregnant and lactating women [100]. Current recommendations from the Food and Drug Administration (FDA) and American Thyroid Association (ATA) in the event of a nuclear emergency are evacuation, sheltering, the avoidance of contaminated food, milk, and water ingestion, and KI prophylaxis of 130 mg daily for the adult population until the risk of significant exposure to ^131^I no longer exists [101].

## 6. Iodine and Cancer

There are reports of anti-proliferative and pro-apoptotic effects of iodine on breast cancer cells based on animal studies. In animal and human studies, molecular iodine supplementation has been shown to have a suppressive effect on tumor growth and size. These effects may occur through direct action, where oxidized iodine dissipates the potential of the mitochondrial membrane, causing mitochondrial apoptosis, and indirectly through iodolipid formation and the activation of gamma-type peroxisome proliferator-activated receptors (PPARγ), which, in turn, triggers apoptotic or differentiation pathways [102]. Iodine also inhibits the formation of nitric oxide (NO) in mouse macrophages and the expression of tumor necrosis factor-α (TNFα) in human monocytes/macrophages [103].

Some researchers suggest that there is a link between iodine deficiency and human mammary gland disease [104]. Clinical studies have shown that molecular iodine has a beneficial effect on fibrotic breast disease [105]. Women suffering from breast cancer show a significant decrease in the excretion of iodine; therefore, it is expected that iodine may play a significant role in the differentiation and integrity of breast cells. Previous studies show that iodine deficiency also results in hyperplasia of the breast tissue and its hypertrophy, as well as peri-alveolar and ductal fibrosis [106]. The impact of thyroid diseases on the development of breast cancer is well documented [107]. Abnormal thyroid function may promote breast cancer through the action of thyroid hormones and their stimulation of αvβ3 integrin receptor inducing mitogen-activated protein kinase (MAPK) activity and the estrogen receptors pathway response; the interaction of TSH and TSH-stimulating antibodies in the course of Graves’ disease on the extra thyroid receptors for TSH (TSH-R) localized in breast tissue; and the action of an increased concentration of prolactin (PRL) accompanying the primary hypothyroidism. However, hypothyroidism and the presence of thyroid autoantibodies may be protective against breast cancer, reducing its incidence and progression. Recent reports suggest that a normal concentration of iodine in the body also protects against breast cancer, but there are no epidemiological studies on the individual risk of developing the disease. It has been noted that, in areas where exposure to both selenium and iodine is high (Japan), the risk of breast cancer is lower than in areas where selenium levels are high and iodine levels are low, or in areas where the concentration of both elements is low (Northern Europe) [108].

An interesting observation was made by analyzing the occurrence of breast cancer in the Japanese population. It has been demonstrated that breast cancer is more common in women who either emigrated from Japan or follow a “western” diet, whereas, in women who follow a local diet based on algae and iodine-rich fish, breast cancer is significantly less frequent [109]. It has been shown that this also applies to the occurrence of breast cancer in Japanese men.

In 2005, an attempt was made to determine whether it is possible to identify breast cancer based on differences in urine iodine concentrations [110]. The study was conducted in women with diagnosed breast cancer, as well as in healthy women and women with benign changes in the breasts. The results of the study showed statistically lower levels of iodine in the urine of women with breast cancer, regardless of age (lower concentrations in premenopausal women) and smoking (lower concentrations in smokers).

In rats subjected to four-week iodine therapy, a comparative assessment of breast cancer tissue in the treatment group showed less vascular tissue, lower levels of tumor proliferation nuclear antigen (PNCA), lower levels of vascular endothelial growth factor (VEGF), lower levels of urokinase plasminogen activator (u-PA), and lower levels of peroxisome alpha proliferator-activated receptors (PPARα) than in the control group [111]. The same iodine-treated tumors also showed higher concentrations of apoptosis markers such as caspase 3 and PPARγ and a greater number of terminal deoxynucleotidyl transferase deoxyuridine triphosphate (dUTP) nick end labeling (TUNEL)-positive cells.

In another study, an estrogen-positive (ER+) breast cancer cell culture was treated with Lugol’s solution (5% Iodine (I_2_), 10% KI) [112]. A microarray analysis showed the stimulating effect of the therapy on the expression of 29 genes and its suppressive effect on 14 other genes. The real time polymerase chain reaction (RT-PCR) method confirmed the impact of iodine on the increase in mRNA concentrations of the genes responsible for estrogen metabolism (cytochrome P450 1A1 (CYP1A1), cytochrome P450 1B1 (CYP1B1), aldo-keto reductase family 1C1 (AKR1C1)) and the decrease in the concentration of genes regulated by estrogen (trefoil factor 1 (TFF1), wnt-1-induced signaling protein-2 (WISP2)). The authors of this study suggested that iodine therapy should be tested as a potential adjuvant treatment for women with luminal breast cancer. This hypothesis was verified in clinical settings [113].

Similarly, povidone-iodine (PVP-I) showed a cytotoxic effect on colon cancer cells and ascites cancer cells. The growth of breast cancer and seven other human malignant cell lines was restricted to varying degrees by I_2_ and iodolactones. Although KI itself did not affect cell proliferation, it enhanced the anti-proliferative effect of I_2_. PVP-I significantly inhibited the proliferation of michigan cancer foundation-7 (MCF-7) human breast cancer, induced pluripotent cancer (IPC) melanoma, and A549 and H1299 lung cancer cells at a concentration corresponding to 20 µM I_2_. Similarly, Lugol’s solution at concentrations corresponding to 20–80 µM I_2_ decreased the growth of MCF-7 cells. Experiments with fresh human blood samples showed that the anti-proliferative activity of PVP-I and I_2_ is highly conserved in plasma. These results suggest that PVP-I, Lugol’s solution, and the combination of iodide and I_2_ could be powerful agents for the development of anti-cancer strategies [114].

Iodine also holds promise for improved treatment in patients with brain metastases [115]. It is estimated that they occur in approximately 30% of advanced breast cancer patients. Studies on aggressive human breast cancer growth in the brains of athymic nude mice have led to the development of iodine nanoparticles (INPs), which, when injected intravenously, show a predilection for metastasis in the CNS. Accumulated iodine in the tumor then absorbs X-rays during radiotherapy (RT), creating free radicals that increase both the local dose of RT in the tumor and local damage to the tumor. Initial treatment with INP resulted in long-term remissions with 40% of treated mice surviving 150 days and 30% surviving >280 days. With radiotherapy alone, the survival rate was up to 72 days [115]. These reports show the possible benefits of the use of iodine in the diagnosis and treatment of breast cancer.

Iodine deficiency may also be associated with a higher risk of developing stomach cancer. It is hypothesized that iodine deficiency (or excess) may be a risk factor for gastric cancer and atrophic gastritis. This effect of iodides on the gastric mucosa may be due to antioxidant activity and antagonism to iodide inhibitors such as nitrates, thiocyanates, and salt, which are risk factors for gastric carcinogenesis [116]. Tissue iodine levels, as determined by the Foss method based on the Sandell–Kolt–Hoff reaction, were lower in gastric cancer tissue compared to surrounding normal tissue. There was a positive correlation between iodine levels in gastric cancer tissue and the surrounding normal tissue. Therefore, iodine deficiency may be one of the factors behind the increased incidence of gastric cancer [117]. A study in Iran compared the urinary-iodine-to-urine-creatinine ratio in 100 patients diagnosed with stomach cancer and 84 controls. The mean urine iodine level was lower in gastric cancer patients (61.9 µg/g creatinine, compared to 101.7 µg/g creatinine in the control group (*p* < 0.0001)), and more cancer patients (49.0%) had severe iodine deficiency (<25 µg/g creatinine) than control patients (19.1% (*p* < 0.0001)). This study concluded that the relationship between stomach cancer and iodine deficiency is significant [118]. In other studies, the relationship was also observed between the improvement in the iodine supply and a decrease in the incidence of gastric cancer, which may indicate a protective role of iodine prophylaxis in iodine-poor areas [119]. 

However, epidemiological research conducted on the Thai population between 1990 and 2009 showed a possible negative effect of the iodine fortification of salt on carcinogenesis. Although it was observed that the implementation of iodine supplementation correlated to a decrease in the incidence of goiter and follicular cancer, it was found to be related to an increase in the incidence of papillary thyroid cancer [120]. These observations were significant in previously highly iodine-deficient areas. 

Additional research is needed to better understand the effect of iodine on carcinogenesis and the clinical outcome of iodine deficiency compensation in cancer patients.

## 7. Prevalence of Iodine Deficiency

Data from 2021 show that iodine deficiency occurs in all regions of the world and affects countries at every stage of economic development [121]. The implementation of salt iodization has significantly reduced the effects of iodine deficiency worldwide in recent years, and the few reported iodine deficiencies are mild to moderate [30]. Excess iodine is found in 13 countries in the world, mainly due to excess groundwater iodine levels or over-iodized salt supplementation. Iodine consumption is the lowest in Madagascar. There are also shortages in Vietnam and Cambodia. Regional iodine deficiencies concern Russia and Sudan. An insufficient iodine intake, caused by war or natural disasters, is also reported in Haiti and Iraq. Iodine deficiencies also exist in countries with highly developed medical care systems, including Norway, Germany, and Finland [30]. Scandinavian countries, i.e., Norway, Sweden, and Denmark are also struggling with insufficient iodine nutrition during pregnancy [122].

Iodine prophylaxis introduced in Poland in 1997 eliminated the deficiency of this element in the population. According to the recommendations of the Polish Society of Endocrinology, women planning pregnancy and pregnant or breastfeeding women should consume an additional dose of iodine. However, a 2015 study found that only 45% of pregnant women take the recommended daily dose of iodine [123]. As many as 21 European countries struggle with the problem of iodine deficiency in pregnancy, 23 countries lack data, and only 10 countries have sufficient iodine levels [124]. Obese women are particularly exposed to the deficiency of iodine and other micronutrients during pregnancy due to the use of diets rich in energy but low in essential micronutrients [125]. It has been hypothesized that they should become the key target of iodine supplementation [126]. Hypothetically, an increased fat intake may interfere with iodine absorption and the release of pro-inflammatory cytokines from adipose tissue, and insulin resistance may be responsible for a decrease in the expression of the sodium–iodide symporter in enterocytes, resulting in a reduction in iodine absorption [127].

The growing problem of obesity may suggest that the threat of iodine deficiency will be even more significant. The data on urinary iodine levels in overweight and obese children are inconsistent and show both increased and decreased levels when compared to normal-weight children [128]. 

An interesting and extremely important report in the context of determining the norm of iodine concentration in urine for pregnant women is the multinational analysis carried out by Wong et al. [129]. When the median urinary iodine concentration (UIC) in school-age or non-pregnant women indicated that the iodine intake was adequate or above the requirement, approximately half of the pregnant women showed an insufficient iodine intake [129]. These findings indicate that further research should also include measurements of iodine excretion in this particularly sensitive group.

Iodine intake is closely related to the food iodine content and dietary changes. It is estimated that, in the European countries and the US, the milk and dairy contribution of the recommended daily iodine intake for adults ranges between 13–64% [130]. In the UK, dairy products were one of the main sources of iodine in the diet. Until 2011, it was believed that the iodine deficiency had been eliminated. As a result of the conducted studies, iodine deficiency was detected in school-age children, especially in those who consumed small amounts of milk [131]. By measuring the iodine content of products consumed by Libyans, it has been shown that bread is a significant source of iodine in Libya. This is mainly due to the addition of iodized salt to the dough. The measured iodine content in wheat flour ranged from 39 to 48 µg [132]. This is due to the dependence of the Libyan economy on imported grain. The amount of iodine in bread, pasta, couscous, and rice is correlated with the place of its origin. The variable factor is the soil and the ways of fertilizing and irrigating it, which are different in different parts of the world. The culture of consuming algae and seaweed, which is especially rich in iodine, is widespread in Japan. It is estimated that the daily iodine intake of Japanese adults is 1–3 mg/day [133]. Spices are another major source of iodine. The average daily consumption of spices by the Libyan population is 6 g, which is approximately 7.4 µg/day of iodine [132]. For comparison, the average person in India consumes approximately 25 g of spices per day [134]. It is worth noting that heating food for 30–90 min at and above 100 °C causes a significant loss of iodine, as it becomes volatile at temperatures above 58 °C. Iodine losses during various cooking processes, e.g., frying, grilling, and cooking, are estimated at 20%, 23%, and 58%, respectively [135].

Iodine deficiency may also develop in patients on diets low in iodine-rich foods and restricted in salt. This applies, inter alia, to patients with a risk of cardiovascular disease. The 2015 guidelines of the Polish Society of Hypertension (PTNT) recommend limiting table salt to 5 g/day due to the hypertensive and pro-atherosclerotic effect of a sodium chloride (NaCl)-rich diet [136]. At the same time, it is emphasized that a high supply of table salt contributes to the pathogenesis of strokes and some cancers [137]. A reduction in salt consumption to the recommended 5 g/day of table salt may decrease the iodine intake to less than the recommended 150 µg per day [138]. Alternative iodine enrichment options have been considered in some cases where a dietary salt restriction is sought. It has been shown that iodine enrichment in food, drinks, spices, and additives other than salt probably increases the concentration of iodine in the excreted urine, which indirectly reflects its level in the body [139]. The most advanced iodine fortification programs enrich natural iodine carriers such as milk, mineral water, and even plants [130,140,141]. An adequate iodine intake can also be achieved by iodine enrichment in animal (i.e., cattle) food, resulting in a higher iodine content in milk and meat.

The alternative solution is an increase in iodine salt fortification coordinated with the monitoring of salt and iodine intake levels [142].

## 8. Supplementation and Fortification

Thirty-two countries, whose citizens account for almost one-third of the world’s population, still report an insufficient iodine intake, with a significant risk of iodine deficiency in pregnancy [143,144]. Socioeconomic factors may play a role in non-compliance with iodine supplementation programs. A study in Italy found that poverty and a lack of access to public health resulted in the restricted use of iodized salt and iodine supplements for poor and immigrant women [145]. Another factor contributing to the aggravation of iodine deficiency in the population is the limitation of the iodized salt intake as a general recommendation. On the other hand, a greater availability of products and spices from around the world may increase the supply of iodine other than through iodized salt.

In Australia, New Zealand, and Russia, iodized bread has been introduced to supplement iodine [146,147]. Water seems to be a very promising vehicle for iodine fortification, as it is accessible to poor and isolated persons and has been introduced in many different countries, such as Mali, Malaysia, and China [148,149,150]. However, water iodization is more costly than salt iodine fortification. 

The optimal daily dose of iodine (150–250 µg/day for an adult) allows for the minimization of the amount of thyroid dysfunction in the general population. In most countries, the best strategy to reduce the deficiency is controlled salt iodination. This appears to be the most economical and effective way [151]. On the basis of epidemiological studies, the Polish Commission for the Control of Iodine Deficiency Disorders (PCCIDD) has determined the remaining indications for additional prophylaxis of iodine deficiency, which includes iodination of the initial and subsequent nutrition for newborns (10 μg per 100 mL of milk) and additional supplementation in pregnant and lactating women with an additional dose of iodine of 150–200 μg/day [12,152]. Iodine supplementation seems to be of particular importance in the case of pregnancy. Therefore, many medical societies around the world, such as ATA, European Thyroid Association (ETA), or American Academy of Pediatrics (AAP), now recommend iodine supplementation for women who are pregnant, breastfeeding, or planning pregnancy [153,154,155]. Ideally, iodine supplementation should be started at least three months before conception to ensure that the mother has an adequate supply of iodine in the thyroid gland. The success of maternal iodine supplementation varies by region. A low effectiveness was observed in regions where educational activities aimed at the general public were ineffective [152,156,157].

## 9. Conclusions

Iodine deficiency is an ongoing problem. Pregnant women, breastfeeding women, and children are particularly affected by iodine deficiency. It leads to thyroid diseases and metabolic and developmental disorders, as well as other diseases, including oncological ones. Correcting an iodine deficiency potentially reduces the chance of developing malignancies. Urine iodine levels should be monitored more frequently and an increase in iodine salt levels should be considered.

To monitor iodine nutrition and classify the iodine world status, nationwide representative studies, usually in children 6–12 years of age, should be performed with urine iodine measurements every three years. This school population is readily available and representative of the general population. Research should also include measurements of iodine excretion in particularly sensitive groups, such as pregnant women, as the adequate iodine nutrition status of school-age children or nonpregnant women may not indicate an adequate iodine nutrition status among pregnant women.

The upcoming public health challenge appears to be reducing salt consumption, which could result in a lower iodine intake. Thus, an iodine enrichment vehicle other than salt should be commonly introduced.

## Figures and Tables

**Table 1:** Assessment of iodine nutrition based on urinary iodine concentrations (UIC) and grades of iodine deficiency according to World Health Organization (WHO) [28].

Age Group	UIC (µg/L)						
	Excessive	More than Adequate	Adequate	Inadequate	Mild Insufficiency	Moderate Insufficiency	Severe Insufficiency
adults and school-age children	≥300		100–299	<100	50–99	20–50	<20
pregnant women	≥500	250–499	150–249	<150			
lactating women			≥100	<100			

## Data Availability

Not applicable.
